# Examining the emotional impact and financial strain on caregivers of those with Duchenne muscular dystrophy in Saudi Arabia: findings from a questionnaire-based cross-sectional study

**DOI:** 10.3389/fpsyg.2026.1828571

**Published:** 2026-06-18

**Authors:** Yazed Alruthia, Azzam Alobaysi, Horiyah Alhajri, Ghadah Almuaythir, Hala H. Alrasheed, Lama Mohammed Alqarni, Ahmed Khamis Bamaga

**Affiliations:** 1 Pharmacoeconomics Research Unit, College of Pharmacy, Riyadh, Saudi Arabia; 2 Department of Clinical Pharmacy, College of Pharmacy, King Saud University, Riyadh, Saudi Arabia; 3 Corporate Department of Pharmacy Services, King Saud University Medical City, Riyadh, Saudi Arabia; 4 Saudi Food and Drug Authority, Riyadh, Saudi Arabia; 5 College of Medicine, King Abdulaziz University, Jeddah, Saudi Arabia

**Keywords:** caregivers, Duchenne, financial stress, muscular dystrophy, quality of life, Saudi Arabia

## Abstract

**Background:**

Duchenne muscular dystrophy (DMD) is a severe, progressive, and life-limiting neuromuscular disorder that imposes a substantial economic burden on healthcare systems and families. While the clinical severity of DMD is well established, limited data exist regarding its socioeconomic and psychological impacts in Saudi Arabia. This study investigated patient and caregiver demographics, direct out-of-pocket expenditures, caregiver depression and anxiety, and health-related quality of life (HRQoL).

**Methods:**

A descriptive, cross-sectional, questionnaire-based telephone survey was conducted over a 12-month period, from September 2023 to September 2024 among caregivers recruited from outpatient neurology clinics at a university-affiliated tertiary care center in Riyadh, Saudi Arabia. Data were collected using validated Arabic-language instruments, including the Costs for Patients Questionnaire (CoPaQ) for expenditures, the 9-item Patient Health Questionnaire (PHQ-9) for depression, the 7-item Generalized Anxiety Disorder (GAD-7) scale for anxiety, and the EuroQol 5-Dimension 5-Level (EQ-5D-5L) for HRQoL.

**Results:**

The study included 40 informal caregivers, predominantly fathers (57.5%) and males (57.5%), with a mean age of 42 years. Most participants (77.5%) did not have private health insurance. Families incurred significant financial strain, with a mean total out-of-pocket expenditure of US$ 12,400 ± 14,070.27, driven primarily by prescription medications, annual medical travel, and home modifications. Low-income families were disproportionately affected. Caregivers experienced considerable psychological distress, as indicated by a mean PHQ-9 score of 8.18 and a mean GAD-7 score of 7.2. Bivariate linear regression showed that greater depression severity (*p* = 0.0003), greater anxiety severity (*p* < 0.0001), the presence of caregiver chronic health conditions (*p* = 0.0034), and caring for more DMD patients (*p* = 0.0008) were significantly associated with lower EQ-5D-5L utility scores. In contrast, higher monthly income (*p* = 0.0029) and male gender (*p* = 0.0454) were significantly associated with higher utility scores.

**Conclusion:**

Informal caregiving for children with DMD in Saudi Arabia frequently results in severe psychological distress and substantial out-of-pocket expenses. Assessment of HRQoL and caregiver mental health highlights an urgent need for national clinical pathways, enhanced physician education, and comprehensive, family-centered public health interventions.

## 1 Background

### 1.1 Pathophysiology and diagnosis of Duchenne muscular dystrophy

Duchenne muscular dystrophy (DMD) is a severe, progressive, and life-limiting neuromuscular disorder. It is an X-linked recessive genetic condition resulting from mutations, primarily large deletions, duplications, or point mutations, in the DMD gene. These mutations severely impair or prevent the production of dystrophin protein (Alghamdi et al., 2021). Dystrophin is a critical cytoskeletal protein that protects and stabilizes muscle fibers during contraction. Its absence results in ongoing muscle necrosis, inflammation, and eventual replacement of muscle tissue with fibrotic and adipose tissue (Łoboda and Dulak, 2020).

Clinical manifestations usually appear between ages three and five, including proximal muscle weakness, calf pseudohypertrophy, toe walking, and a positive Gowers’ sign. Disease progression leads to further motor decline, lumbar lordosis, scoliosis, and, in some cases, intellectual impairments or neurodevelopmental delays (Sinha et al., 2017). Without intervention, ambulation is typically lost by age 12, and patients face life-threatening cardiopulmonary complications in late adolescence or early adulthood. While robust Saudi-specific mortality data for DMD is currently lacking, a knowledge gap that further underscores the urgent need for a comprehensive national registry (Bamaga et al., 2021; Aldharee, 2024), local cause-of-death patterns are largely assumed to mirror these global cardiopulmonary trends (Andrews and Wahl, 2018).

Early and accurate diagnosis is essential for optimizing care and delaying functional decline. Initial screening generally involves measuring serum creatine kinase (CK) levels, which are markedly elevated in DMD patients (Tavakoli et al., 2023). If CK levels are elevated, genetic sequencing is recommended to identify specific mutations (Tavakoli et al., 2023). When genetic testing is inconclusive, which occurs in approximately 25–30% of cases, muscle biopsy is necessary to assess dystrophin protein levels and mRNA transcripts (Alghamdi et al., 2021; Elbashir et al., 2022).

### 1.2 Global and regional epidemiology

Globally, the estimated prevalence of muscular dystrophy is 3.6 per 100,000 individuals, with the Americas reporting the highest prevalence at 5.1 cases per 100,000 individuals (Salari et al., 2022). The epidemiological landscape of DMD in the Middle East, particularly in Saudi Arabia, presents distinct challenges (Aldharee, 2024).

Saudi Arabia currently lacks a comprehensive national registry for DMD, yet clinical experts report a significant familial recurrence rate. High consanguinity rates and larger median family sizes in the region often result in multiple male children with DMD within a single household (Bamaga et al., 2021). Additionally, Saudi Arabia’s demographic history, shaped by migrations from Asia, Africa, and Europe, has produced distinct high-frequency deletion regions (HFDRs) within the DMD gene among the Saudi population, necessitating tailored genetic screening approaches (Tayeb, 2010). Despite these factors, delayed diagnosis remains a critical issue. Early non-motor symptoms, such as delayed speech, are frequently misdiagnosed, postponing definitive diagnosis and the initiation of pharmacological and rehabilitative care by up to 30 months (AlSaman et al., 2022; Plejic and Kuzmanic Šamija, 2022). This delay not only severely hampers the timely initiation of corticosteroids and vital rehabilitation but also postpones access to essential genetic counseling for family planning (AlSaman et al., 2022). Consequently, this diagnostic prolonged limbo dramatically increases caregiver uncertainty and emotional strain, heavily compounding the initial trauma of the diagnosis (Landfeldt et al., 2014; Uttley et al., 2018).

The epidemiological reality of DMD in Saudi Arabia translates directly into a profound multidimensional burden for families. As the disease clinically progresses, marked by the predictable loss of ambulation and creeping respiratory decline, parents face exponentially increasing caregiving hours and chronic sleep disruption (Landfeldt et al., 2018; Furnback et al., 2022; Schwartz et al., 2022). The relentless uncertainty surrounding the patient’s prognosis and functional milestones fosters deep anxiety and anticipatory grief. Ultimately, the child’s functional decline permeates every aspect of daily life, severely affecting parental mental health, overall family functioning, and the ability to participate socially (Uttley et al., 2018; Furnback et al., 2022). Therefore, understanding this emotional domain is just as critical as addressing the clinical and economic challenges.

### 1.3 Disease management and healthcare gaps

Current therapeutic strategies for Duchenne muscular dystrophy (DMD) are mainly palliative, focusing on symptom management and maintaining ambulatory function (Yao et al., 2021). Long-term use of glucocorticoids like prednisone and deflazacort is standard for reducing inflammation and preserving muscle strength, but they can lead to significant side effects, such as weight gain and osteoporosis (Zhang and Kong, 2021). Although novel gene therapies show promise, their accessibility is limited (Elbashir et al., 2022).

In the Middle East and North Africa (MENA) region, clinical practice for DMD management remains inconsistent. While highly advanced services and multidisciplinary care are available in select tertiary centers across Saudi Arabia, the country still lacks standardized, consistent access to these comprehensive care models nationwide (Bamaga et al., 2021, 2023). This situation highlights the urgent need for national clinical pathways, improved physician education, and integrated telerehabilitation protocols to support home-based physical therapy (Saaei and Klappa, 2021; AlSaman et al., 2022).

### 1.4 Economic burden and out-of-pocket expenditures

DMD imposes a substantial and escalating economic burden on healthcare systems and families as functional dependency rises (Landfeldt et al., 2014; Furnback et al., 2022; Katsomiti et al., 2025). Shehata et al. (2023) found that while direct medical costs, such as physiotherapy, medications, and mobility aids, remain relatively constant across disease stages, non-medical and indirect costs, particularly informal care provided by family members, constitute the majority of the economic burden, reaching up to 54% of total costs in later stages. For families, out-of-pocket expenditures for home modifications, specialized transportation, private physical therapy, and uninsured medical equipment can be financially devastating (Schwartz et al., 2022). However, data specific to the Saudi Arabian context remain limited.

### 1.5 Psychosocial impact and quality of life

The progressive nature of DMD significantly affects the health-related quality of life (HRQoL) of both patients and their families (Uttley et al., 2018; Katsomiti et al., 2025). For patients, the loss of independent ambulation and increasing reliance on caregivers substantially diminishes quality of life during the second decade of life (Uttley et al., 2018).

The psychological impact on caregivers is also significant but often under-researched (Landfeldt et al., 2018). The chronic and demanding nature of informal caregiving for a child with DMD frequently results in severe psychological distress (Landfeldt et al., 2018). Caregivers are at increased risk for clinical depression, anxiety, and social isolation due to the combined pressures of financial strain, anticipatory grief, and daily physical demands.

In Saudi Arabia, this psychosocial impact is distinctly shaped by regional cultural dynamics. Gendered caregiving expectations often place the daily physical and emotional burden disproportionately on mothers (Alshammari et al., 2023; Almulla et al., 2024), while the high prevalence of consanguineous marriages can introduce complex family dynamics, including internalized or perceived blame. Furthermore, families may face social stigma associated with severe hereditary conditions. Conversely, these challenges are often navigated through strong religious beliefs that foster acceptance and resilience, alongside robust extended family networks that can serve as a critical buffer to mitigate the intense isolation and burden of caregiving (Alshammari et al., 2023; Almulla et al., 2024). Assessing the HRQoL and mental health status of caregivers is essential for developing comprehensive, family-centered public health interventions.

### 1.6 Study rationale and objectives

Despite the recognized clinical severity of DMD, there is a significant lack of data regarding its socioeconomic and psychological impacts within Saudi Arabia. To address these gaps in the literature and inform regional public health policy, this study has four primary objectives:

1. To describe the demographic and clinical characteristics of patients with DMD in Saudi Arabia.
2. To examine the direct out-of-pocket expenditures incurred by families caring for patients with DMD.
3. To assess the prevalence and severity of depression and anxiety among informal caregivers.
4. To evaluate the overall health-related quality of life (HRQoL) of these caregivers.

## 2 Materials and methods

### 2.1 Study design and setting

A descriptive, cross-sectional, questionnaire-based telephone survey was conducted over a 12-month period. The study population was recruited from outpatient neurology clinics at a university-affiliated tertiary care center in Riyadh, Saudi Arabia.

### 2.2 Study population and sampling

The target population consisted of informal caregivers of patients with a confirmed diagnosis of DMD residing in multiple regions of Saudi Arabia. Participants were recruited through convenience sampling. Caregivers were contacted by telephone and enrolled after providing informed consent.

*A priori* power analysis was conducted using G*Power version 3.1 to detect a point-biserial correlation (r_*pb*_) with an effect size of 0.5 between key variables (e.g., HRQoL utility scores, depression and anxiety scores, and total out-of-pocket expenditures) (Faul et al., 2007). An effect size of 0.5 was selected based on Cohen’s standard conventions for representing a large effect in behavioral and psychological research (Cohen, 2013), anticipating a strong correlation between the intensive demands of DMD caregiving and subsequent socioeconomic and mental health outcomes. Assuming an alpha level of 0.05 and a power of 80%, the analysis indicated that a minimum sample size of 26 caregivers was required to achieve statistical significance.

### 2.3 Inclusion and exclusion criteria

Participants were selected based on the following criteria:

- Inclusion criteria: Primary caregivers of patients with a confirmed diagnosis of DMD who had a disease history of at least one year, and who possessed a registered, active telephone number.
- Exclusion criteria: Caregivers of patients whose DMD diagnosis was established less than one year prior to the study. Additionally, cases involving patients under 18 years of age where a legally authorized guardian was unavailable to provide proxy consent were excluded.

### 2.4 Data collection and instruments

Data collection was conducted by four trained researchers using structured telephone interviews, each lasting approximately 20 min. The survey incorporated a set of validated Arabic-language instruments to address the study’s primary objectives:

1. Sociodemographic and clinical characteristics: Data collected included the age and gender of both patients and caregivers, family annual income, private supplemental health insurance coverage, patient educational level, and family history of DMD or other neurological conditions. The Arabic version of the Single Item Literacy Screener (SILS), validated by Al-Jumaili et al. (2015), was utilized to evaluate health literacy (Morris et al., 2006). Participants rated their need for assistance reading medical materials on a 5-point scale (1 = Never to 5 = Always). Scores ≥3 were classified as indicating limited health literacy (Morris et al., 2006; Al-Jumaili et al., 2015).
2. Out-of-pocket expenditures: The Arabic version of the Costs for Patients Questionnaire (CoPaQ) was utilized to comprehensively capture direct and indirect medical costs, as well as any non-medical expenses directly related to managing the disease (Laberge et al., 2021; Alotaibi et al., 2024).
3. Caregiver mental health: The Arabic version of the 9-item Patient Health Questionnaire (PHQ-9) was utilized to evaluate depression severity (range: 0–27). Following established scoring guidelines, PHQ-9 scores were classified as indicating minimal (0–4), mild (5–9), moderate (10–14), moderately severe (15–19), or severe (20–27) depression (Kroenke et al., 2001; AlHadi et al., 2017). The Arabic version of the 7-item Generalized Anxiety Disorder (GAD-7) scale was utilized to evaluate anxiety severity (score range: 0–21) (Sawaya et al., 2016). Following established scoring guidelines, GAD-7 scores were classified as indicating minimal (0–4), mild (5–9), moderate (10–14), or severe (15–21) anxiety (Spitzer et al., 2006).
4. Health-related quality of life (HRQoL): Caregiver quality of life was assessed using the validated Arabic version of the EuroQol 5-Dimension 5-Level (EQ-5D-5L) instrument (Al-Jedai et al., 2024). Moreover, the utility scores were calculated using the country-specific value set for the Kingdom of Saudi Arabia, as established by Al-Jedai et al. (2024).

### 2.5 Ethical considerations

The study protocol received Institutional Review Board (IRB) approval. Verbal informed consent was obtained from all caregivers, including those acting as proxies for minor patients, prior to the interviews. Participants were informed about the study’s purpose, risks, benefits, and their right to withdraw at any time. Strict privacy and confidentiality standards were implemented for genetic and medical information, with data access limited to authorized research personnel. In recognition of the potential psychosocial burden related to DMD, researchers conducted interviews with sensitivity and ensured impartial access to participation for all eligible individuals.

### 2.6 Statistical analysis

All statistical analyses were performed using SAS software, version 9.4 (SAS Institute Inc., Cary, NC, USA). Descriptive statistics summarized the baseline characteristics of the study sample: continuous variables were reported as means with standard deviations (SD), and categorical variables as frequencies and percentages. Inferential statistics, including Student’s *t*-tests, one-way analysis of variance (ANOVA), Fisher’s exact tests, Chi-square tests, and Spearman’s rank correlation, were applied as appropriate to assess associations between variables. Bivariate linear regression was conducted to examine the relationship between different sociodemographic characteristics of the caregivers and the HRQoL utility based on the EQ-5D-5L. A *p*-value less than 0.05 was considered statistically significant.

## 3 Results

### 3.1 Sociodemographic and clinical characteristics

The study sample comprised 40 informal caregivers of patients diagnosed with DMD, with a mean age of 42 years. Most respondents were male (57.5%) and the patients’ fathers (57.5%). The cohort was predominantly Saudi (92.5%), with the largest proportion residing in the Makkah region (47.5%). Notably, 77.5% of caregivers lacked private health insurance, and 22.5% reported a monthly household income of less than $1,333. The average age of the patients was 12 years (mean illness duration: 5.15 years). While most caregivers managed a single affected patient (77.5%), 22.5% cared for two or more. High rates of consanguinity (42.5%) and familial history of DMD (45%) were reported. Detailed sociodemographic and clinical characteristics are summarized in Table 1.

**TABLE 1:** The characteristics of DMD patients’ caregivers (*n* = 40).

Characteristic	*N* (%)
Age	
30–35 years	10 (25)
36–40 years	9 (22.50)
41–45 years	9 (22.50)
46–50 years	7 (17.50)
> 50 years	5 (12.50)
Gender	
Male	23 (57.5)
Female	17 (42.5)
Region	
Riyadh	11 (27.5)
Makkah	19 (47.5)
Madinah	2 (5)
Eastern region	1 (2.5)
Asir	2 (5)
Al-Jouf	1 (2.5)
Tabuk	3 (7.5)
Northern borders	1 (2.5)
Relationship to DMD patients	
Mother	16 (40)
Father	23 (57.5)
Sister	1 (2.5)
Marital status	
Married	35 (87.5)
Divorced	4 (10)
Widowed	1 (2.5)
Number of affected DMD patients in the family	
One	31 (77.50)
Two	6 (15)
Three	3 (7.5)
Patients age	
5–10 years	17 (42.5)
11–15 years	15 (37.5)
> 15 years	8 (20)
Nationality	
Saudi	37 (92.5)
Non-Saudi	3 (7.5)
Education	
Elementary school	2 (5)
Intermediate school	2 (5)
High school	11 (27.5)
Associate degree	5 (12.5)
Bachelor’s degree	15 (37.5)
Postgraduate degree	5 (12.5)
Is there any consanguinity between the parents?	
Yes	17 (42.5)
No	23 (57.5)
Does either parent have any relatives with Duchenne muscular dystrophy?	
Yes	18 (45)
No	22 (55)
Health literacy	
Adequate	34 (85)
Limited	6 (15)
Comorbidities	
Hypertension	10 (25)
Depression	5 (12.5)
Diabetes	8 (20)
Dyslipidemia	3 (7.5)
Social anxiety disorder	1 (2.5)
Monthly income	
< $1,333	9 (22.50)
1,333–$2,667	14 (35)
2,667–$4,000	10 (25)
> $4,000	7 (17.5)
Private health insurance coverage	
Yes	9 (22.5)
No	31 (77.5)

### 3.2 Caregiver mental health outcomes

**FIGURE 1:**
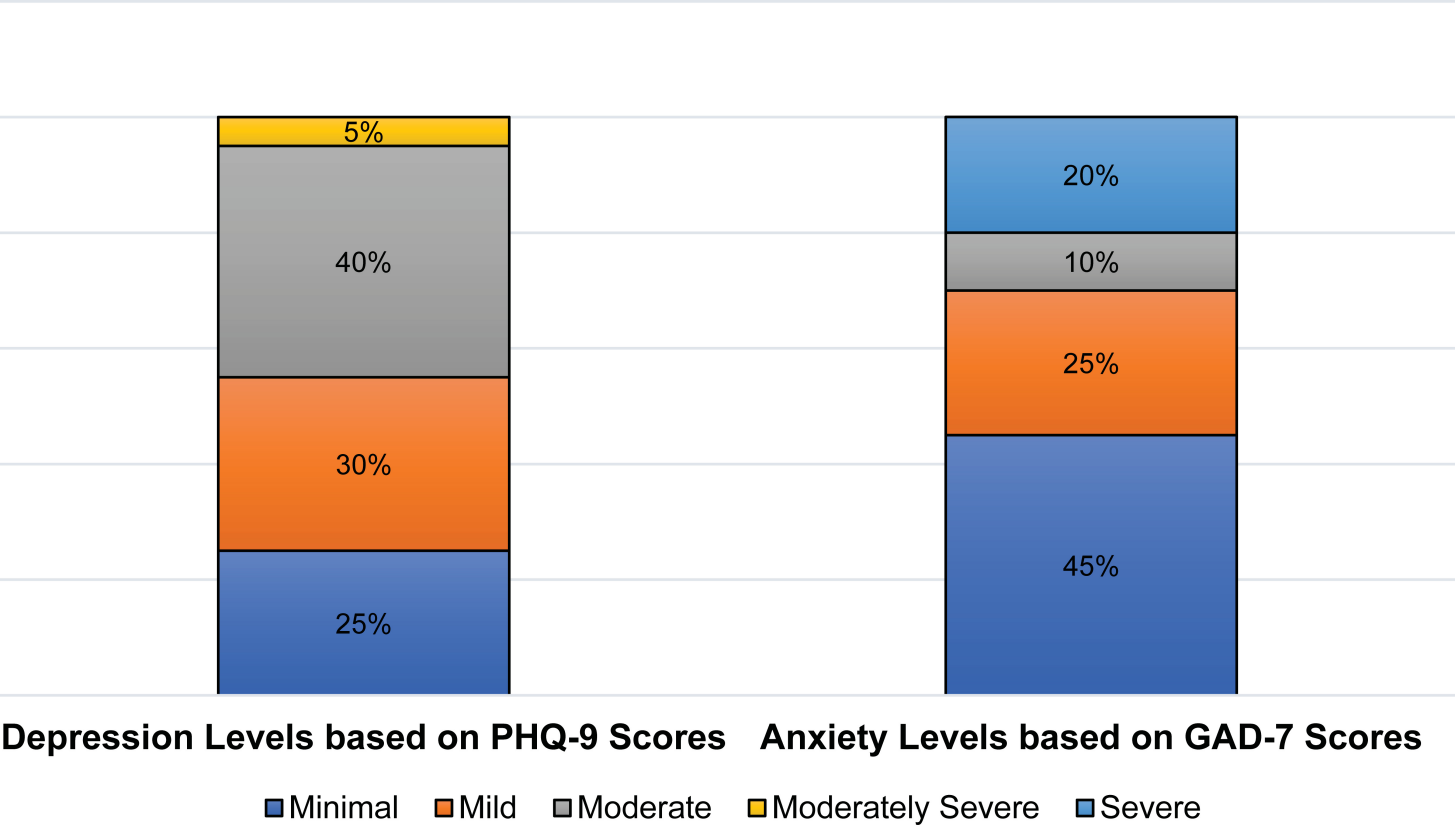
The levels of depression and anxiety among patients’ caregivers based on PHQ-9 and GAD-7 scores.

To understand the effects of caregiving, the study assessed psychological impact using the PHQ-9 and GAD-7 instruments (Figure 1). Caregivers had a mean PHQ-9 score of 8.18, indicating a notable burden of depressive symptoms. Mild depression was present in 30% of respondents, while 40% experienced moderate depression.

Additionally, assessment of anxiety symptoms yielded an average GAD-7 score of 7.2. While nearly half of the participants (45%) reported experiencing minimal anxiety as shown in Figure 1.

### 3.3 Financial burden and out-of-pocket expenditures

**FIGURE 2:**
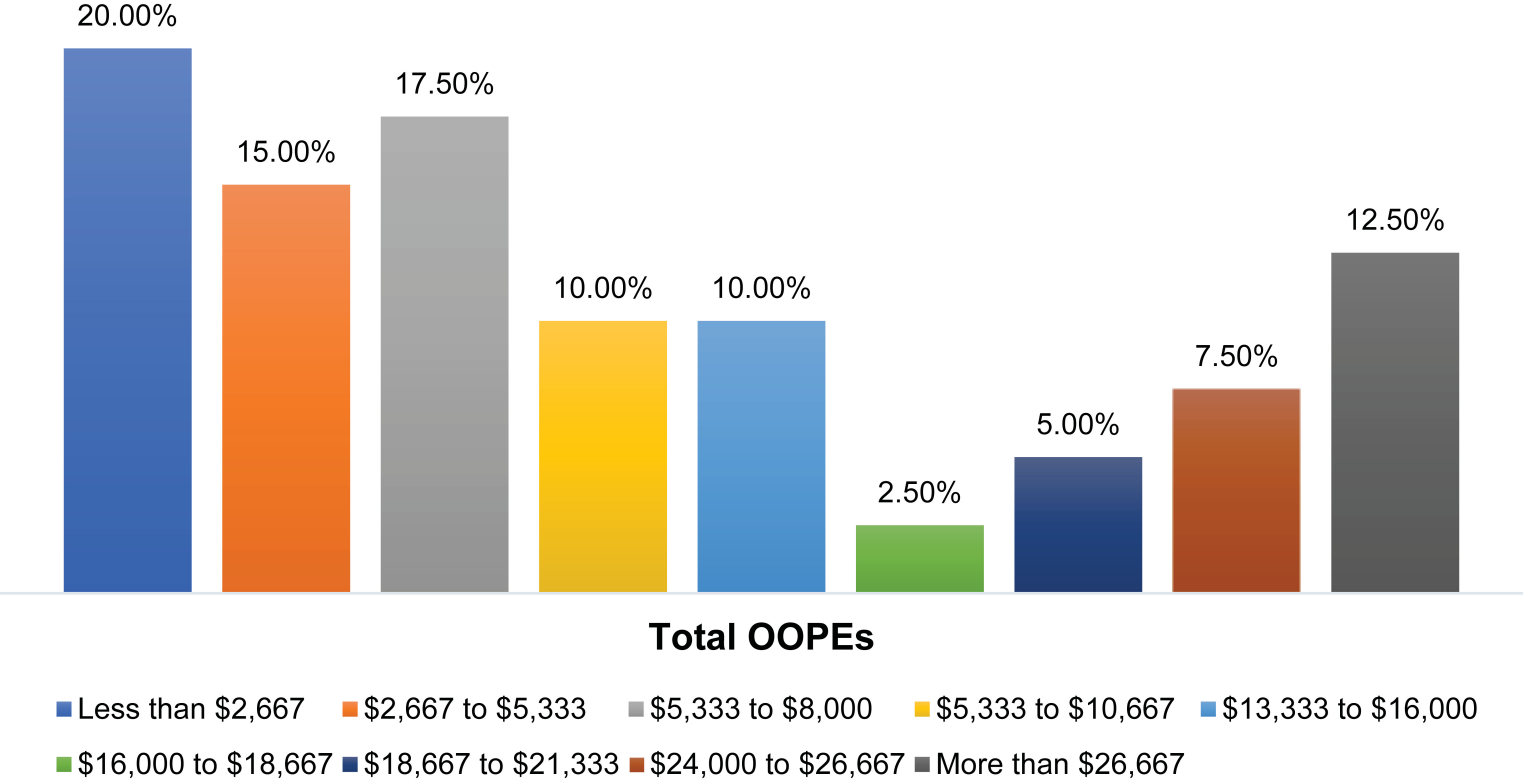
Estimated total OOP expenditures since DMD diagnosis.

The economic strain on families was substantial, characterized by significant out-of-pocket (OOP) expenditures. Families faced direct and indirect costs for managing the disease, which included expenditures for prescription medications, annual travel for medical care, medical devices and physical therapy, and home or vehicle remodeling for health reasons. The breakdown of these estimated annual expenditures is summarized in Table 2. These combined financial obligations resulted in a heavy economic burden, as shown in Figure 2. Importantly, these expenses for medications and transportation were found to disproportionately affect low-income families within the cohort.

**TABLE 2:** Estimated annual OOPEs across different cost categories.

Cost category	Mean cost (US$) ± SD	Median cost (US$) [IQR]	Percentage contribution to total annual OOPEs
Prescription medications	465.58 ± 632.06	342.86 [700]	11.51%
Travel	591.63 ± 611.34	422.22 [707.69]	14.63%
Medical devices/physical therapy	1,537.27 ± 3,263.96	535.71[962.35]	38.02%
Home/vehicle remodeling	721.99 ± 989.32	342.86 [807.69]	17.85%
Others (e.g., alternative medicine)	727.27 ± 431.26	800.00 [857.14]	17.99%
Total OOPE	4,042.69 ± 4,379.80	2,552.38 [2,775.76]	

### 3.4 Caregiver health-related quality of life (HRQoL)

**FIGURE 3:**
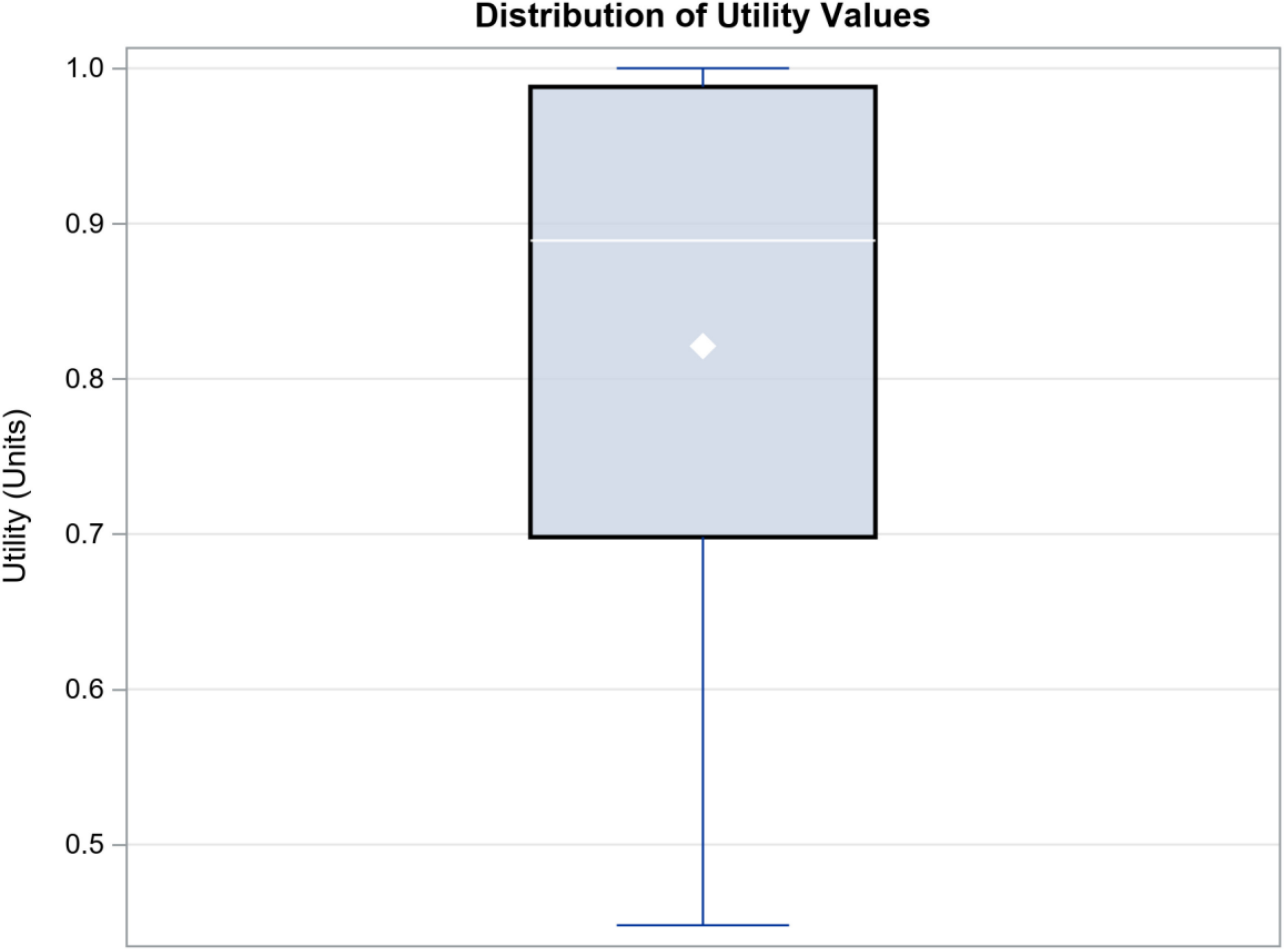
The utility scores of the DMD patients’ caregivers.

Caregiver health-related quality of life was measured using the EuroQol-5D-5L (EQ-5D-5L) instrument across five domains (Table 3). A large majority reported no problems with mobility (72.5%) or self-care tasks such as washing or dressing (97.5%). While 55% had no difficulties with usual activities, 12.5% were entirely unable to perform their daily routines. Regarding pain and discomfort, 55% reported no pain, yet 25% reported moderate symptoms. Anxiety and depression assessments showed that 42.5% reported no symptoms in this domain; on the other hand, 22.5% indicated moderate, and 15% severe or extreme levels. Figure 3 presents overall utility scores derived from the EQ-5D-5L.

**TABLE 3:** EuroQol-5-D-5-L scores for DMD patients’ caregivers.

EQ-5D-5L domains	*N* (%)
Mobility	
I have no problems in walking about	29 (72.5)
I have slight problems in walking about	7 (17.5)
I have moderate problems in walking about	2 (5)
I have severe problems in walking about	2 (5)
I am unable to walk about	0 (0)
Self-care	
I have no problems washing or dressing myself	39 (97.5)
I have slight problems washing or dressing myself	0 (0)
I have moderate problems washing or dressing myself	0 (0)
I have severe problems washing or dressing myself	1 (2.5)
I am unable to wash or dress myself	0 (0)
Usual activities	
I have no problems doing my usual activities	22 (55)
I have slight problems doing my usual activities	7 (17.5)
I have moderate problems doing my usual activities	3 (7.5)
I have severe problems doing my usual activities	3 (7.5)
I am unable to do my usual activities	5 (12.5)
Pain/discomfort	
I have no pain or discomfort	22 (55)
I have slight pain or discomfort	8 (20)
I have moderate pain or discomfort	10 (25)
I have severe pain or discomfort	0 (0)
I have extreme pain or discomfort	0 (0)
Anxiety/depression	
I am not anxious or depressed	17 (42.5)
I am slightly anxious or depressed	8 (20)
I am moderately anxious or depressed	9 (22.5)
I am severely anxious or depressed	3 (7.5)
I am extremely anxious or depressed	3 (7.5)

### 3.5 Regression analysis of EQ-5D-5L utility scores

A bivariate linear regression examined the association between EQ-5D-5L utility scores and sociodemographic and clinical factors (Table 4). Several variables were significant predictors of health-related quality of life (HRQoL). Male caregivers had higher utility scores than female caregivers (β = 0.11508, *p* = 0.0454). Higher monthly income was also significantly associated with higher utility scores (β = 0.07984, *p* = 0.0029).

**TABLE 4:** Bivariate linear regression analyses of the relationship between utility scores and sociodemographic characteristics, as well as PHQ-9 and GAD-7 scores.

Characteristic	β estimate	95% confidence limits		*p*-value
		Lower	Upper	
Age	−0.00782	−0.01594	0.00030371	0.0587
Gender (male vs. female)	0.11508	0.00250	0.22766	0.0454
Number of DMD patients the guardian cares for	−0.15004	−0.23355	−0.06653	0.0008
Number of years since patient diagnosis	0.00468	−0.01102	0.02038	0.5498
Education	0.01778	−0.02531	0.06087	0.4089
Income	0.07984	0.02900	0.13068	0.0029
Health literacy (limited vs. adequate)	0.09269	−0.06738	0.25275	0.2484
Presence of chronic health conditions	−0.17460	−0.28786	−0.06133	0.0034
PHQ-9 score	−0.02365	−0.03553	−0.01176	0.0003
GAD-7 score	−0.02043	−0.02662	−0.01424	< 0.0001
Total OOPEs	0.01385	−0.00376	0.03145	0.1197

OOPEs, out-of-pocket expenditures.

Conversely, several factors negatively impacted caregiver HRQoL. An increase in the number of DMD patients cared for by the guardian was significantly associated with lower utility scores (β = −0.15004, *p* = 0.0008). Chronic health conditions among caregivers also significantly reduced utility scores (β = −0.17460, *p* = 0.0034). Worse mental health was a strong negative predictor: greater depression severity on the PHQ-9 (β = −0.02365, *p* = 0.0003) and greater anxiety severity on the GAD-7 (β = −0.02043, *p* < 0.0001) both significantly correlated with lower utility scores. Other variables, including caregiver age (*p* = 0.0587), education level (*p* = 0.4089), years since patient diagnosis (*p* = 0.5498), health literacy (*p* = 0.2484), and total Out of Pocket Expenditures (OOPEs) (*p* = 0.1197), were not significantly associated with utility scores in this model.

## 4 Discussion

This cross-sectional study highlights the profound emotional and financial burdens shouldered by informal caregivers of patients with DMD in Saudi Arabia. Despite the high prevalence of familial recurrence (45%) and consanguinity (42.5%) observed in our cohort, caregivers remain highly vulnerable to out-of-pocket (OOP) financial strain and significant psychological morbidity. Specifically, our findings demonstrate that caring for multiple affected children, coupled with a lack of private health insurance, acts as a major catalyst for diminished caregiver health-related quality of life (Bamaga et al., 2021; Aldharee, 2024).

A key finding is the substantial financial burden DMD places on caregivers. This impact threatens both family economic stability and wider societal productivity. Families reported large out-of-pocket (OOP) costs for prescription medications, medical travel, specialized devices, and home or vehicle modifications according to the CoPaQ. The substantial financial burden identified in our study—driven largely by prescription medications, medical travel, and home modifications—is exacerbated by the fact that 77.5% of our cohort lacked private health insurance. This aligns with findings by Shehata et al. (2023) in Egypt, who noted that non-medical costs and informal caregiving constitute up to 54% of the economic burden. Additionally, our results align with other findings from Greece and worldwide: disability, specialized transport, and uninsured medical equipment all contribute to the financial strain on caregivers (Landfeldt et al., 2018; Katsomiti et al., 2025). However, our data uniquely highlights the systemic gaps in the Saudi context, where specialized transport and uninsured medical equipment disproportionately decimate the financial stability of low-income families.

Psychologically, the mean PHQ-9 (8.18) and GAD-7 (7.2) scores in our cohort indicate that 70% of caregivers are navigating mild to moderate depression. Caregivers of children with DMD often experience significant psychological distress, anticipatory grief, and social isolation (Uttley et al., 2018; Shehata et al., 2023). These findings support systematic reviews that note the chronic under-support of caregiver burden in DMD (Furnback et al., 2022). The Single Item Literacy Screener (SILS) showed that most caregivers had adequate health literacy (Morris et al., 2006). However, tailored medical communication remains essential for effective disease management.

Regression analysis of EQ-5D-5L utility scores revealed factors influencing caregivers’ quality of life. Higher monthly income and being male were associated with higher utility, suggesting that financial security helps protect against the disease’s socioeconomic impact. In contrast, a greater number of DMD patients in the household, caregiver health issues, and more severe depression and anxiety predicted lower utility scores. When compared to the utility scores of the general Saudi population reported by Kroenke et al. (2001), the significantly lower utility scores among our caregivers underscore the severe, isolating effects of chronic disease management (Al-Jedai et al., 2024).

Solving these clinical and socioeconomic challenges requires systemic changes in Saudi Arabia’s ongoing healthcare transformation. Delayed diagnoses are a key issue. Non-motor symptoms, such as delayed speech, are often misdiagnosed. Without prompt newborn screening, genetic sequencing, or muscle biopsies, the initiation of essential therapies is dangerously delayed (Bamaga et al., 2021). Glucocorticoids like prednisone and deflazacort can delay motor decline (Zhang and Kong, 2021). However, they also cause severe side effects such as osteoporosis. As patients age and lose ambulation, they develop fatal cardiopulmonary complications, which makes seamless transition care vital (Yao et al., 2021; Zhang and Kong, 2021).

In the Middle East and North Africa (MENA), clinical care pathways are often fragmented (Bamaga et al., 2021, 2023; Aldharee, 2024). Access to new gene therapies is limited (Bamaga et al., 2021). A shortage of pediatric neurologists in Saudi Arabia means general pediatricians frequently care for these complex cases (Bamaga et al., 2023). To improve societal productivity and caregiver outcomes, the healthcare system should adopt standardized, multidisciplinary care models. Adding telerehabilitation can support home-based physical therapy and reduce travel and financial burdens for families. Finally, family-centered public health programs are crucial for reducing the negative effects of DMD and ensuring fair care amid the country’s healthcare reforms.

This study provides insights into the socioeconomic and psychological impacts of Duchenne muscular dystrophy in Saudi Arabia, though several limitations exist. First, its cross-sectional design collects data at a single point in time, preventing conclusions about causality between caregiving demands and health or mental health outcomes. Second, convenience sampling occurred at one university-affiliated tertiary care center in Riyadh. While caregivers lived across multiple regions and the final cohort of 40 exceeded the required 26, the small, single-center sample may limit generalizability. Third, self-reported data from 20-min telephone interviews using tools like the CoPaQ, PHQ-9, and GAD-7 may introduce recall bias, especially regarding historical out-of-pocket expenses. Finally, excluding caregivers of patients diagnosed less than one year before the study may omit data reflecting the immediate financial and psychological impact of diagnosis.

## 5 Conclusion

This study highlights the profound burden of Duchenne muscular dystrophy (DMD) on informal caregivers in Saudi Arabia. The disease’s progressive and life-limiting course severely affects patients. It also imposes substantial financial and psychological strain on their families. Caregivers reported significant out-of-pocket expenses for prescription medications, medical devices, and travel related to the disease. These costs disproportionately impacted low-income households and those without private health insurance. Beyond financial hardship, caregiving demands led to considerable psychological morbidity. Caregivers experienced high rates of depression and anxiety. Regression analysis showed that greater severity of depression and anxiety, lower household income, and more affected family members strongly predicted lower HRQoL for caregivers.

To reduce negative impacts on productivity and family wellbeing, these findings underscore the urgent need for change. Saudi Arabia should align DMD management with the ongoing healthcare transformation. Comprehensive national registries and standardized multidisciplinary care models are essential. Integrating solutions like telerehabilitation is also a critical public health priority. Addressing the severe economic and mental health challenges caregivers face will help policymakers. They can promote a more equitable, family-centered healthcare system and improve the quality of life for individuals affected by DMD.

## Data Availability

The original contributions presented in this study are included in this article/supplementary material, further inquiries can be directed to the corresponding author.
